# A single-arm feasibility cohort study of rivaroxaban in antiphospholipid syndrome

**DOI:** 10.1186/s40814-020-00594-1

**Published:** 2020-04-25

**Authors:** Kimberly Legault, Mark Blostein, Marc Carrier, Susan Khan, Sam Schulman, Sudeep Shivakumar, Cynthia Wu, Mark A. Crowther

**Affiliations:** 1 Department of Medicine, McMaster University, 1280 Main St. W, Hamilton, ON L8S 4L8 Canada; 2 Department of Medicine, McGill University, 845 Sherbrooke St. W., Montreal, QC, H3A 2T5 Canada; 3 Department of Medicine, University of Ottawa, 75 Laurier Ave E, Ottawa, ON K1N 6N5 Canada; 4 Department of Medicine, Dalhousie University, 6299 South St, Halifax, NS B3H 4R2 Canada; 5 Department of Medicine, University of Alberta, 116 St & 85 Ave, Edmonton, AB T6G 2R3 Canada

**Keywords:** Antiphospholipid syndrome, Feasibility studies, Hemorrhage, Rivaroxaban, Thromboembolism

## Abstract

**Background:**

There is uncertainty regarding the safety and effectiveness of direct oral anticoagulant agents in patients with antiphospholipid syndrome (APS). We performed a multicenter feasibility study to examine our ability to identify and obtain consent from eligible APS patients and to obtain 95% adherence with daily rivaroxaban administration, in order to inform and power a larger study. Clinical outcomes of bleeding and thrombosis were also collected.

**Methods:**

APS patients with prior venous thromboembolism (VTE) were recruited over 2 years (Oct 2014–Sept 2016) and followed for ≥ 1 year. Patients were assessed clinically every 3 months and had pill counts performed every 6 months. Numbers of patients fulfilling study criteria, as well as those consenting to participate, were tracked, and percentage adherence based on pill counts was recorded. These data were compared against the feasibility endpoints. Rates of thrombosis and bleeding were calculated. Criterion for feasibility was obtaining consent from 135 of 150 identified APS patients over 2 years.

**Results:**

Ninety-six eligible patients were identified, and 14 declined participation. Eighty-two patients were followed for a mean of 19 months, representing 129.8 patient-years. Average rivaroxaban adherence was 95.0%. During follow-up, there were 4 thromboembolic events (2 cerebrovascular and 2 VTE). There were no episodes of major bleeding.

**Conclusions:**

Adequately powered comparative trials using patient-important outcomes in APS are unlikely to be successful due to inability to recruit sufficient numbers of study subjects. This study does not reveal a higher than expected risk of recurrent thromboembolic disease compared to historical cohorts; however, this is an uncontrolled study in relatively low-risk APS patients.

**Trial registration:**

The study was registered with clinicaltrials.gov, identifier NCT02116036, April 16, 2014.

## Introduction

The antiphospholipid syndrome (APS) is characterized by clinical manifestations (venous or arterial thrombosis and/or recurrent pregnancy loss) occurring in patients with persistent antiphospholipid antibody positivity [either anticardiolipin antibody (aCL), lupus anticoagulant (LA, otherwise known as non-specific inhibitor), and/or anti-beta-2 glycoprotein-1 antibody (aβ2GP1), positive on ≥ 2 occasions ≥ 12 weeks apart] [1]. APS is a common cause of myocardial infarction and stroke in patients under the age of 50 and is particularly prevalent in patients with autoimmune conditions, especially systemic lupus erythematosus (SLE) [2]. The prothrombotic state associated with APS may cause either arterial or venous thrombosis, with about 55% of reported events being either arterial or mixed arterial and venous [3]. Our multicenter collaborative research group has confirmed that the optimal therapy for the prevention of recurrent venous or arterial thrombosis in patients with APS is warfarin administered to achieve an international normalized ratio (INR) of 2.0–3.0 [4].

Although highly effective, warfarin requires frequent, careful monitoring [5]. Conversely, direct oral anticoagulants (DOACs) are replacing warfarin for many indications in Canada due to comparable efficacy for indications including prevention and treatment of venous thromboembolism (VTE) and stroke prevention in patients with nonvalvular atrial fibrillation, with comparative ease of use [6–9].

However, in patients with APS, simple extrapolation from other clinical situations (where these agents are safely substituted for warfarin) is not warranted given the unique characteristics of this population. First, some patients with APS seem to be at very high risk for recurrent thrombosis, suffering a higher than expected rate of recurrent events despite “usual intensity” therapy with warfarin [10, 11]. Second, many patients with APS receive medications such as corticosteroids, azathioprine, anti-inflammatory medications, or other agents that can either directly or indirectly influence the risk of thrombosis and bleeding and which may interact with one of the these newer agents, increasing or decreasing drug levels [5, 12]. This is particularly important as there is no reliable method of monitoring DOAC therapy. Third, patients with APS are different than those enrolled in the clinical trials that have demonstrated the efficacy of the DOACs; they are generally younger and have both chronic and acute comorbid conditions which may influence the risk of both first and recurrent thrombosis (e.g., concomitant inflammatory disorders, immobilization, renal insufficiency, and the need for surgical procedures). Taken in sum, these observations suggest that before DOACs are widely adopted in patients with APS, we require good quality evidence for their efficacy and safety.

Given the uncertainty regarding the safety and efficacy of DOACs in patients with APS, we initiated a research program dedicated to this question. A large definitive study powered to detect clinically important outcomes would be required to address this question; however, prior to mounting such a large and expensive study, we carried out a pilot feasibility study. Our rationale for a pilot study was as follows: APS is a rare disease and eligible consenting patients may be difficult to identify and enroll despite the participation of 6 clinical centers; patients with APS may have a strong preference for continuing to receive warfarin given the evidence of benefit from previous studies; and adherence, if suboptimal, may pose safety considerations given the potential increased risk of thrombosis in the setting of short-term interruptions in rivaroxaban administration. A pilot study would thus allow for a more accurate estimate of enrollment capacity and consent rates, provide information regarding expected adherence, and allow for an estimate of rates of thrombosis and bleeding to inform a larger definitive study.

In this pilot feasibility study, we explored the following feasibility outcomes: (a) we examined our ability to identify 150 eligible APS patients; (b) we measured our ability to obtain consent from 135 of these patients; and (c) we tested our hypothesis that we could obtain 95% adherence with daily rivaroxaban administration.

## Material and methods

Adult (≥ 18 years) patients fulfilling criteria for APS [1] who had prior VTE and who did not have a contraindication to use of rivaroxaban were recruited to participate at 6 clinical sites in Canada (St. Joseph’s Healthcare Hamilton, Hamilton, ON; Hamilton Health Sciences, Hamilton, ON; Jewish General Hospital, Montreal, QC; QEII Health Sciences Centre, Halifax, NS; The Ottawa Hospital, Ottawa, ON; and University of Alberta Hospital, Edmonton, AB) over a 2-year period (October 2014–September 2016). Exclusion criteria included previous recurrent thrombosis while taking warfarin, need for therapy with either rivaroxaban 2.5 mg or 5 mg BID or both aspirin and clopidogrel for acute coronary syndrome (ACS), pregnancy, chronic renal insufficiency with glomerular filtration rate < 30, geographic inaccessibility, inability to provide informed consent, or situation where, in the opinion of the treating physician, rivaroxaban is to be avoided (e.g., excessive bleeding risk, hypersensitivity), or another anticoagulant is preferred (e.g., mechanical heart valve, active malignancy). In addition, as rivaroxaban 20 mg daily is not approved in Canada for secondary prevention of thrombosis in patients with ACS or stroke, patients with isolated arterial thromboembolism as the sole manifestation of APS were also excluded.

All successfully recruited patients were followed for a minimum of 1 year (follow-up period completed September 2017). If patients were eligible to participate but declined consent, the reason for declining participation was collected, if possible. All consenting participants were transitioned from their current anticoagulant to rivaroxaban 20 mg orally daily. Patients were reassessed clinically every 3 months by telephone or in-person visits to collect data on thrombotic events, bleeding events, or other adverse events. In-person visits occurred at minimum every 6 months, when pill counts were completed to determine adherence. Informed consent was obtained from all participants, and research ethics board approval for the study was obtained from each clinical site (Hamilton Integrated Research Ethics Board identifier for central site: #14-366). Independent adjudicators assessed thromboembolic and bleeding events. An independent safety monitor reviewed all adverse events. Two levels of safety monitoring were built in—informal consecutive patient monitoring and formal analyses after 50 and 100 recruited patients (if reached) achieved 6 months of follow-up. Given that the estimated frequencies of both thrombosis and bleeding from previous warfarin studies were 3–6%, the upper limit of 6% was chosen as the safety limit for this study. The frequencies of adjudicated thrombotic and bleeding events were separately calculated with a 97.5% confidence interval (using significance of 0.05 for the set of tests and Bonferroni correction applied for two independent tests) at the two monitoring levels during the study. Using this method, 10 bleeding or thrombotic events would have to have been recorded for the first 50 patients, or 14 for the first 100 patients, to trigger a safety concern. The study was registered with clinicaltrials.gov, identifier NCT02116036. The pilot trial protocol can be accessed from the corresponding author upon request.

### Outcome measures

#### Primary outcomes (feasibility objectives)

- Enrolment—We defined success as the ability to identify 150 eligible patients over the study period.
- Consent—We defined success as a consent rate of 90% (thus, with identification of 150 patients, at least 135 would have provided consent).
- Compliance—We defined success as missing fewer than 5% of days with pill administrations.

#### Secondary outcome measures

- Bleeding—rates of major and minor bleeding were collected.
  - Major bleeding was defined using International Society of Thrombosis and Haemostasis criteria [13]
  - Minor bleeding was defined as all bleeding that did not meet criteria for major bleeding
- Thrombosis—rates of VTE and arterial thrombosis were collected. Outcome definitions for VTE, myocardial infarction, ischemic stroke, and cardiovascular death were adapted from the COMPASS study [14].

Data was recorded through Research Electronic Data Capture (REDCap) [15]. Data analysis was performed by comparing recruitment rates to the pre-specified feasibility endpoints and by calculating rates of thrombosis and bleeding events. The sample size for the study, i.e., 135 patients followed for a minimum of 1 year, was chosen to allow us to obtain estimates of bleeding and thrombosis rates in addition to the assessments of recruitment rates and adherence. The rationale was that if recruitment had occurred as planned, the average follow-up of enrolled patients would be about 16 months, providing about 180 patient-years of exposure. This exposure was expected to be sufficient to accrue between 4 and 8 adjudicated bleeding and thrombotic events, allowing us to calculate estimates of bleeding and thrombosis rates with reasonable 95% confidence intervals.

## Results

### Included patients

Ninety-six patients meeting inclusion/exclusion requirements were identified over the 2-year period from October 2014 to September 2016, and 14 patients declined participation (85% consent rate). Fourteen of 96 eligible patients declined consent, with the reasons stated being desire to remain on warfarin therapy (7/14), logistical (e.g., difficulty getting to study center for study visits, 2/14), or no clear stated reason (e.g., “not interested,” 5/14). The resulting 82 patients were followed for a mean of 19.0 months (median 21 months), ending September 2017, representing 129.8 patient-years of data. Baseline characteristics are described in Table 1; 10 patients (8.2%) had SLE. Forty-seven patients (57.3%) were single-positive for antiphospholipid antibodies (APLA) (i.e., had one of LA, aCL, or aβ2GP1 positive), 11 patients (13.4%) were double-positive (i.e., had two of LA, aCL, and/or aβ2GP1 positive), and no patient was triple-positive (i.e., were LA, aCL, and aβ2GP1 positive). Notably, 24 of the patients in the study were found at analysis not to have fulfilled the Sapporo criteria for persistent APLA positivity, having either had only one isolated positive result, or aCL and/or aβ2GP1 at low-titer only, though all patients were considered to have APS by the treating clinician.

**Table 1:** Baseline demographics of included patients

Demographic	*N* (%) or result
Female sex	39 (47.6%)
Average age at study entry	53.4 years
Previous arterial events	5 (6%)
≥ 2 previous thromboses	24 (29.2%)
On warfarin at study entry	41 (50%)
On rivaroxaban at study entry	38 (46.3%)
LA positive	46 (56%)
aCL positive	30 (36.6%)
aβ2GP1 positive	4 (4.9%)
Double positivity	11 (13.4%)
Triple positivity	0 (0%)
SLE	10 (8.2%)

*aCL* anticardiolipin, *aβ2GP1* anti-β2 glycoprotein-1, *LA* lupus anticoagulant, *SLE* systemic lupus erythematosus

### Medication adherence

Fifty-five patients (67%) had pill counts performed on at least one occasion. One of these patients had unusable data due to the use of inpatient supply during hospitalization; thus, data from 54 patients were used for adherence determination. Forty-four of 54 patients (81%) achieved > 95% adherence. Average adherence was 95.0%.

### Clinical outcomes

Over the follow-up period, there were 4 recurrent thromboembolic events (2 arterial cerebrovascular events, 1 deep vein thrombosis, and 1 pulmonary embolus) (3.0 events/100 patient-years) in patients taking rivaroxaban. Characteristics of patients experiencing a recurrent event are described in Table 2. There were two thrombotic episodes in patients who were no longer taking rivaroxaban at the time of the event: One patient had VTE while receiving low molecular weight heparin after development of hepatitis secondary to disseminated histoplasmosis necessitating discontinuation of rivaroxaban, and a second patient had ischemic stroke while on warfarin after having rivaroxaban discontinued due to headache and hypertension. None of the patients who experienced recurrent thromboses had SLE.

**Table 2:** Characteristics of patients with recurrent thrombotic event

Characteristic	Patient 1	Patient 2	Patient 3	Patient 4
Location of thrombus	MCA stroke	PE	MCA stroke	DVT
Age	33 years	19 years	64 years	54 years
Sex	F	M	M	F
aPL profile	LA	LA	aCL, titer 23	LA
Number of previous thromboses at baseline	4	1	2	4
Prev arterial thrombosis?	Y	N	N	N
SLE?	N	N	N	N
> 95% adherence?	Y	Y	Y	No bottles returned

*aCL* anticardiolipin antibody, *aPL* antiphospholipid antibody, *DVT* deep venous thrombosis, *MCA* middle cerebral artery, *LA* lupus anticoagulant, *PE* pulmonary embolism, *SLE* systemic lupus erythematosus

There were 23 episodes of minor bleeding, and no episodes of major bleeding. In 3 of these episodes, specific medical intervention was required to control bleeding. Eight of these patients had rivaroxaban held temporarily, and one patient discontinued study drug.

Thirty-two patients had a total of 45 reported adverse events during the study, though there were no serious adverse events (Table 3). Ten of these adverse events were drug-related or possibly drug-related. Nine events required permanent discontinuation of study medication. A further 6 patients withdrew consent partway through the study or were lost to follow-up.

**Table 3:** Adverse events

Adverse Event(s)	Rivaroxaban-related? (n=)	Rivaroxaban discontinued? (n=)
Infectious (Influenza, respiratory tract, fever NYD, cellulitis, pneumosepsis, disseminated histoplasmosis, viral infection NOS) n=9	n=0	n=1
Gastrointestinal (Nausea, melena, colonoscopy, 20 lb weight gain, epigastric pain) n=8	n=2	n=1
Headache n=5	n=4	n=2
Dermatological (Rash, finger nodules, leg ulcer) n=4	n=1	n=2
Neurological (Peripheral neuropathy/CNS impairment NYD, Bell’s palsy) n=3	n=0	n=0
SLE flare (including class IV lupus nephritis) n=2	n=0	n=1
Other (Chest pain, broken tooth, right hip arthroplasty, back pain, depression, cough, post-thrombotic syndrome, felt unwell, joint pain, prostate cancer, hypertensive urgency, SOB NYD) n=10	n=0	n=2
Death (Pneumosepsis) n=1	n=0	N/A

Full study data can be accessed upon request from the corresponding author. CONSORT Extension to Pilot and Feasibility Trials checklist is attached as Additional file 1.

## Discussion

The study did not meet its feasibility endpoints, with only 96 of a predicted 150 patients identified and 82 of a predicted 135 patients consenting to participate in the study (representing an 85% consent rate, less than the 90% predicted a priori), despite the participation of 6 clinical centers in Canada with expertise in APS. The inclusion only of patients with a history of venous thromboembolic manifestations led to exclusion of patients with isolated arterial events (in Canada, rivaroxaban 20 mg daily is off-label both for cerebrovascular and cardiovascular events; thus such patients were ineligible for the study), which may have affected enrolment appreciably given that in some cohorts arterial events are more common than venous events in patients with APS [16]. Future studies could consider recruitment of patients with a history of isolated arterial events which may improve feasibility of this study design. The two most commonly cited reasons for patients declining to participate in the study were logistical issues, including concern regarding travel to the study center for visits, and patient concern regarding comparative efficacy of the study medication versus warfarin leading them to opt to remain on warfarin. It is difficult to modify the study protocol appreciably to address either of these concerns; from a logistical point of view, the study required in-person visits only every 6 months—a longer time interval such as annual visits could be considered, however would be likely to further affect the adherence measurements, where missing data were already an important concern.

The study technically met its adherence data endpoint where average adherence using the available pill count data was 95.0%; however, there is a risk of bias on this estimate given the amount of missing data for this outcome. No patient had a complete pill count performed for the duration of the study, generally due to patients only returning a portion (usually the last 3 months’ worth) of bottles at each 6-month in-person visit, and a full one-third of the study subjects never returned any bottles at all. However, even with higher rate of return of study bottles, pill counts have been shown to be an imperfect measure of adherence [17, 18]. More robust methods for such determination could be considered, such as laboratory monitoring of anti-Xa activity or electronic pill bottles that record timing of use, though these typically increase the complexity and expense of a study and future studies must balance these competing interests [19]. Ensuring adherence to rivaroxaban in this population is of importance, as its short half-life poses a risk of under-anticoagulation compared to warfarin.

The study does provide efficacy and safety data on 129.8 patient-years of rivaroxaban in APS, making it one of the larger cohorts reported to date. Demographics of patients in our cohort were relatively similar to those of other cohorts of APS patients: approximately 50% were female, with average age in the early 50’s. There were no unexpected adverse events reported for rivaroxaban, and no episodes of major bleeding in this cohort. While there was no comparator group, the rate of recurrent thromboembolic events falls close to the rates seen in the older randomized controlled trials (RCTs) of warfarin use in APS to date (Table 4) [4, 20]. Notably, however, the two most recent RCTs of warfarin in APS have not demonstrated any thromboembolic events in the warfarin groups, which is somewhat surprising given the high-risk features of most of the enrolled patients [21, 22]. It must be noted that the patients enrolled in our study were at relatively low risk of recurrent thrombosis compared to many in the APS population—no patients had had previous failure of warfarin therapy, no patients were “triple positive,” and 20% of the study sample had only low-titer or one-time positive aCL or aβ2GP1. Of the 4 patients who had recurrent thromboembolism, 3 patients were LA positive, which may reflect the slightly higher risk of thrombosis in patients who have isolated LA positivity compared to those with an aCL or aβ2GP1 antibody [23]. The results from our study are in contrast with the recent study by Pengo et al., where a significant imbalance in the composite endpoint of thromboembolic events, major bleeding, and vascular death was seen between patients receiving rivaroxaban compared to warfarin for “triple positive” patients [11 patients in the rivaroxaban group versus 2 patients in the warfarin group (hazard ratio 6.7, 95% CI 1.5–30.5; *p* = 0.01)] leading to premature study discontinuation [22]. There were 7 arterial events in the rivaroxaban group compared to 0 in the warfarin group, and 4 episodes of major bleeding compared to 2 in the warfarin group. Overall, this study does not reveal a high risk of recurrent thromboembolic disease in patients on rivaroxaban; however, it must be noted that this is likely to be a relatively low-risk cohort of APS patients, and this study did not have a control group.

**Table 4:** Comparison of standardized rates of thrombosis in our study versus previous randomized controlled trials of warfarin in antiphospholipid syndrome

	Our study	Crowther, 2003	Finazzi, 2005	Cohen, 2016	Pengo, 2018
No. of patients (No. with arterial events at baseline)	82 (5)	114 (27)	109 (44)	116 (0)	Warfarin arm: 61 (14)
Length of follow-up	19 months (mean)	2.7 years (mean)	3.6 years (median)	6 months	569 days (mean)
No. of recurrent VTE events (%)	2 (2.4)	5 (4.4)	3 (2.3)	0 (0)	0 (0)
No. of recurrent arterial events (%)	2 (2.4)	3 (2.6)	7 (6.4)	0 (0)	0 (0)
Recurrent thrombotic events/100 patient-years	3.1	2.6	2.5	0	0

Abbreviation: *VTE* Venous thromboembolism

Limitations of the study include (1) inclusion only of the subset of APS patients with venous thromboembolism, excluding the subset with isolated arterial events, which may have affected enrolment; (2) the suboptimal rate of return of study bottles for pill count; and (3) the differing antibody profiles of our patients compared to those in other studies of APS patients, with a high number of patients not fulfilling Sapporo criteria for APS.

In conclusion, this study shows that adequately powered comparative trials using clinical outcomes in APS are unlikely to be successful due to inability to recruit sufficient numbers of study subjects, even when recruiting through multiple centers with specific APS expertise. This study does not reveal a higher than expected risk of recurrent thromboembolic disease compared to historical cohorts; however, it is noteworthy that this is an uncontrolled study in relatively low-risk APS patients.

## Supplementary information

**Additional file 1.** CONSORT 2010 checklist of information to include when reporting a pilot or feasibility trial

## Data Availability

The datasets used and/or analyzed during the current study are available from the corresponding author on reasonable request.

## Abbreviations

aβ2GP1: Anti-beta-2 glycoprotein-1 antibody
aCL: Anticardiolipin antibody
ACS: Acute coronary syndrome
APLA: Antiphospholipid antibodies
APS: Antiphospholipid syndrome
DVT: Deep venous thrombosis
DOAC: Direct oral anticoagulant
INR: International normalized ratio
LA: Lupus anticoagulant
MCA: Middle cerebral artery
PE: Pulmonary embolism
RCT: Randomized controlled trial
SLE: Systemic lupus erythematosus
VTE: Venous thromboembolism
