# Correction to: A single-arm feasibility cohort study of rivaroxaban in antiphospholipid syndrome

**DOI:** 10.1186/s40814-020-00614-0

**Published:** 2020-05-18

**Authors:** Kimberly Legault, Mark Blostein, Marc Carrier, Susan Kahn, Sam Schulman, Sudeep Shivakumar, Cynthia Wu, Mark A. Crowther

**Affiliations:** 1 Department of Medicine, McMaster University, 1280 Main St. W, Hamilton, ON L8S 4L8 Canada; 2 Department of Medicine, McGill University, 845 Sherbrooke St. W, Montreal, QC Canada; 3 Department of Medicine, University of Ottawa, 75 Laurier Ave E, Ottawa, ON Canada; 4 Department of Medicine, Dalhousie University, 6299 South St, Halifax, NS Canada; 5 Department of Medicine, University of Alberta, 116 St & 85 Ave, Edmonton, AB Canada

**Correction to: Pilot Feasibility Stud 6, 52 (2020)**

**https://doi.org/10.1186/s40814-020-00594-1**

Following publication of the original article [1], the authors opted to correct the last name of co-author Susan Kahn from Khan to Kahn.
